# A method for zooming of nonlinear models of biochemical systems

**DOI:** 10.1186/1752-0509-5-140

**Published:** 2011-09-07

**Authors:** Mikael Sunnåker, Gunnar Cedersund, Mats Jirstrand

**Affiliations:** 1 Fraunhofer-Chalmers Research Centre for Industrial Mathematics, 412 88 Gothenburg, Sweden; 2 Department of Biosystems Science and Engineering, ETH Zurich, Switzerland; 3 Competence Center for Systems Physiology and Metabolic Diseases, ETH Zurich, Switzerland; 4 Department of Clinical and Experimental Medicine, Diabetes and Integrative Systems Biology, Linkoping University, 581 85 Linkoping, Sweden; 5 Freiburg Institute of Advanced Sciences, Freiburg University, D79104 Freiburg, Germany; 6 Department of Mathematical Sciences, Gothenburg University, 412 96 Gothenburg, Sweden

## Abstract

**Background:**

Models of biochemical systems are typically complex, which may complicate the discovery of cardinal biochemical principles. It is therefore important to single out the parts of a model that are essential for the function of the system, so that the remaining non-essential parts can be eliminated. However, each component of a mechanistic model has a clear biochemical interpretation, and it is desirable to conserve as much of this interpretability as possible in the reduction process. Furthermore, it is of great advantage if we can translate predictions from the reduced model to the original model.

**Results:**

In this paper we present a novel method for model reduction that generates reduced models with a clear biochemical interpretation. Unlike conventional methods for model reduction our method enables the mapping of predictions by the reduced model to the corresponding detailed predictions by the original model. The method is based on proper lumping of state variables interacting on short time scales and on the computation of fraction parameters, which serve as the link between the reduced model and the original model. We illustrate the advantages of the proposed method by applying it to two biochemical models. The first model is of modest size and is commonly occurring as a part of larger models. The second model describes glucose transport across the cell membrane in baker's yeast. Both models can be significantly reduced with the proposed method, at the same time as the interpretability is conserved.

**Conclusions:**

We introduce a novel method for reduction of biochemical models that is compatible with the concept of zooming. Zooming allows the modeler to work on different levels of model granularity, and enables a direct interpretation of how modifications to the model on one level affect the model on other levels in the hierarchy. The method extends the applicability of the method that was previously developed for zooming of linear biochemical models to nonlinear models.

## Background

One of the main reasons for the rapid growth of the field of systems biology is that it makes extensive use of mathematical modeling [1-3]. This allows for a better handling of high complexity, which is an inherent property of all living systems. Using modeling, complex hypotheses can be formulated and tested in a more systematic manner than is possible using only biochemical reasoning [4-6]. However, even if one can obtain a detailed model of the system with a high predictive power, the model in itself does not automatically lead to a full understanding of the underlying biochemistry. One should for instance analyze the model to single out its essence, i.e., to identify those parts of the model that can be eliminated, while still preserving the model's crucial behavior. This latter task is referred to as model reduction, and it is the topic of this paper. There is an extensive literature available on the topic of model reduction. However, most of these studies have been done outside the field of systems biology, and since systems biology brings about new types of challenges, reduction of biochemical models is still in its early stages. Traditional engineering approaches like balanced truncation have focused on preserving the input-output profile in an optimal manner, both for linear [7-10], and for nonlinear [11] systems. However, these methods are not suitable for systems biology, because the reduced model has no natural interpretation in itself (nevertheless, some special cases where this problem can be circumvented have been identified [12,13]). This lack of interpretation is a problem because systems biology models are usually developed to help characterizing the dominating parts and structure of the system, and not only to obtain a black-box predictor. Methods have therefore been developed with traditional chemical approaches that are more centered on reducing the internal dynamics of the system. These methods are typically based on a sensitivity analysis [14-17], on time-scale separation [18-21], or on the lumping of state variables [22-26] (see [20] for a general review on model reduction). The perhaps most widely used method is lumping. Two of the main reasons for this are that an effective lumping scheme can be identified from basic properties of the model (e.g., the stoichiometry), and that lumped state variables are formed as easily interpretable pools of state variables in the original model. However, lumping does normally not come with the possibility of back-translation from the lumped state variables in the reduced model to the original state variables. In [27] we provided such relations. This means that we can take the result from a simulation of a reduced model, and without performing a new simulation, directly compute the corresponding trajectories of the desired original state variables. Because of this back-translation possibility, we refer to the resulting two models as two degrees of zooming of the same model. Nevertheless, like in other recent model reduction papers in systems biology [28-32], the results in [27] were mainly developed with linear systems in mind. Linear systems virtually only appear in the cases of mono-molecular reaction networks and for models describing the probabilistic evolution of a single protein complex [27,33]. However, already in [27] we proposed that zooming may in principle also be applicable to nonlinear models, but we did not derive formulae for back-translation. Note that a majority of the currently available systems biology models are in fact nonlinear.

With the method introduced in this paper, we provide the extension of the previously proposed method in [27] to nonlinear models. We show that new challenges arise due to the nonlinearities, but also how these challenges can be overcome, for instance with a wise choice of state variables in the reduced model. The method is demonstrated by application to two closed models of metabolic systems.

## Methods

In this paper we present a more general version of the method that was introduced in [27], which is applicable to nonlinear models. We start with some basic definitions and key observations that are illustrated on a small example model, before we turn to the details of the method.

### Basic Definitions and Assumptions

The method is developed for models of biochemical reaction systems on state space form that are based on nonlinear ordinary differential equations (ODEs)

(1) ẋ=f(x,p,u,t),

(2) y=h(x,p,u,t),

where *t *denotes time; the dot over *x *in Eq. (1) denotes derivative w.r.t. to time; the state vector *x *∈ ℝ*^n^*; the parameters *p *∈ ℝ*^p^*; the inputs *u *∈ ℝ*^m^*; the outputs *y *∈ ℝ*^l^*; and *f *and *h *are in general nonlinear functions. The state vector, whose individual elements are referred to as state variables, typically represents amounts or concentrations of chemical species, and the parameters commonly represent kinetic constants, initial conditions, or scaling factors. In this paper we are primarily interested in a comparison of the state variables between models (the original model and the reduced model), which means that the form of the nonlinear function in Eq. (2) is irrelevant for the application of our method. The right-hand side of Eq. (1) can be expressed as the stoichiometric matrix *S *∈ ℝ^*n*×*q *^times a vector of reaction rates *r *= *r*(*x*, *p*, *u*, *t*); *r *∈ ℝ*^q^*

ẋ=Sr(x,p,u,t).

The existence of separate time-scales are commonly utilized for reduction of biochemical models (e.g., by reduction of mass action kinetics to Michaelis-Menten kinetics). The typical approach is to investigate if subsets of the state variables are in steady state or in quasi-steady state (QSS). If state variable *x_i _*is in steady-state for *t *≥ 0 it holds by definition that

(3) $${ẋ}_{i}\left(t\right)=0,$$

which implies that

$$x_{i}\left(t\right)=x_{i}\left(0\right),$$

which efficiently removes the state variable from the model, since it can be substituted for constant. If on the other hand the state variable *x_i _*is in QSS, there are terms on the right-hand side of the ODE that are much larger than the negligible term on the left-hand side. The approximation

(4) $$f_{i}\left(x,p,u,t\right)\approx 0.$$

is then commonly used to reduce the model. We refer to a state as fast in the time interval ≤ *T*_0 _≤ *t *<*T*_1 _if Eq. (4) is valid in this time interval, and holds for the class of all considered inputs to the system. Note that *T*_1 _= ∞ in the case that the systems remains in QSS, which may for example not be the case for models with switches (where, e.g., the values of a subset of the state variables may change when a certain condition is fulfilled) [34,35].

Note that Eq. (3) (steady state) necessitates that Eq. (4) (quasi-steady state) is fulfilled, but not *vice versa*. Although QSS implies that (some of) the terms of the right-hand side of the ODE are large and leaves the left-hand side (derivative term) negligible, the derivative term may still be large enough for the state variable in QSS to change considerably during the time-span of a simulation; the key is that these changes mainly occur on a slow manifold.

### Zooming of Linear Models

The concept of zooming was introduced in [27], and a method was presented that is applicable to linear time-invariant (LTI) models, which on state space form reads:

$${ℳ}_{o}:\left\{\begin{array}{c} ẋ=Ax+Bu\\ y=Cx+Du, \end{array}\right)$$

where *A *∈ ℝ^*n *× *n*^, *B *∈ ℝ^*n *× *m*^, *C *∈ ℝ^*l *× *n*^, and *D *∈ ℝ^*l *× *m*^. The method is based on the existence of at least one subset of state variables in ${ℳ}_{o}$ for which the internal dynamics is very fast with respect to the current time-scale of interest. An algorithm for automatic reduction of linear models that is based on the detection of such subsets, which are referred to as fast clusters, is presented in [27]. If the *w *state variables of a fast cluster are replaced by a single state variable $x_{lL}^{\prime }=\sum_{i=1}^{w}x_{l_{i}}$, we obtain a reduced version of the original model

$${ℳ}_{r}:\;\left\{\begin{array}{c} {ẋ}_{r}=A_{r}x_{r}+B_{r}u\\ y=C_{r}x_{r}+Du, \end{array}\right)$$

where *x_r _*∈ ℝ^(*n *- *w *+ 1)^, *A_r _*∈ ℝ^(*n *- *w *+ 1) × (*n *- *w *+ 1)^, *B_r _*∈ ℝ^(*n *- *w *+ 1) × *m*^, and *C_r _*∈ ℝ^*l *× (*n *- *w *+ 1)^.

The fraction parameters, which are typically computed from QSS assumptions and mass conservation relations, take the form

(5) $$\eta_{l_{i}}\left(p\right)=\frac{x_{l_{i}}}{{x^{\prime }}_{lL}},$$

The fraction parameters are used for back-translation of the lumped state variable to the original state variables. Note that the fraction parameters are functions of the model parameters only, and therefore time-invariant; as we will see, these fraction parameter properties do in general not hold for nonlinear models. By comparing the reactions of the original and reduced models, we see that

(6) $$k_{jL}^{\prime }=\overset{w}{\underset{i=1}{\sum }}k_{ji}\eta_{i},$$

where $k_{jL}^{\prime }$ is the rate parameter in the reaction from state variable $x_{lL}^{\prime }$ to state variable *x_j _*in the reduced model, and *k_ji _*is the rate parameter in the reaction from state variable *x_i _*to state variable *x_j _*in the original model.

Finally, note that Eqs. (5) and (6) provide a link between ${ℳ}_{o}$ and ${ℳ}_{r}$, which constitute two different levels of granularity. It is this link between the models that make us consider them as two different degrees of zooming, and the primary goal of this paper is to establish such a link also for nonlinear models.

### Extension to Nonlinear Models - Initial Observations

We will now present some key observations that are used in the derivation of the method for zooming of nonlinear models.

First observe that the mass, which corresponds to a weighted (w.r.t. the molecular weight) sum of the state variables, of a closed (no exchange of matter with the surroundings) nonlinear model is conserved. However, the total number of molecules is in general not conserved in such a model as it is for linear models. This is for example due to the formation and dissociation of complexes, which alters the total number of molecules in the system. For instance, the binding of A to B reduces the number of molecules, as the product AB only counts as one molecule; binding reactions cannot occur in linear models. A second, and related, observation is that another type of conservations appears in nonlinear models; conserved moieties. A moiety is a specific functional part of a molecule, and the weighted sum of the number of molecules that contain this functional part is constant in a closed system. The presence of such a conserved moiety is equivalent to the existence of a row vector *m *∈ ℕ*^n ^*for which *mS *= 0, which also implies that

(7) mẋ=mSr(x,p)=0.

If we let the rank of S be denoted by *n_r_*, the number of linearly independent vectors for which Eq. (7) holds is equal to *n *- *n_r_*, which implies the existence of a matrix *M*

(8) MS=0,

where $M\in {\mathbb{N}}^{n\;-\;n_{r}\;\times \;n}$.

Let us now make some remarks regarding fast state variables in a nonlinear model. Let $x_{f}\in {\mathbb{R}}^{n_{f}}$ be the vector of all fast state variables in *T*_0 _≤ *t *<*T*_1_. For simplification we will assume that there are no inputs to the system, although it would in principle be possible to incorporate inputs in the following discussion. The right-hand side of the ODEs for these fast state variables, if there are no inputs, can be separated into two parts. The first part contains reactions between fast state variables that are significant for the fast dynamics; *r_f _*(*x_f_*, *p*), and the second part contains all other reactions, *r_s_*(*x*, *p*), i.e.,

(9) $${ẋ}_{f}=S_{s}r_{s}\left(x,p\right)+S_{f}r_{f}\left(x_{f},p\right),$$

where *S_f _*and *S_s _*are the corresponding stoichiometric matrices. Let us now consider the fast stoichiometric matrix, *S_f_*, and especially the conserved moieties that are implied by *S_f_*. Since these moieties are only (approximately) conserved on a fast enough time-scale, we refer to such moiety conservations as apparent conservations. Let *M_f _*be a matrix with *a *linearly independent rows such that

(10) $$M_{f}S_{f}\text{ = 0,}$$

where $M_{f}\in {\mathbb{N}}^{a\times n_{f}}$. Each row of this matrix thus implies an apparent conserved moiety in the system. Let the sums of state variables that correspond to apparent moiety conservations (i.e., lumps of state variables) be denoted by *l*, so that

(11) $$l\text{ = }M_{f}x_{f}\text{.}$$

If we differentiate *l *with respect to time, we get

(12) $$\begin{array}{c} \overset{{}^{\circ}}{l}=M_{f}{ẋ}_{f}=M_{f}\left(S_{s}r_{s}\left(x,p\right)+S_{f}r_{f}\left(x_{f},p\right)\right)\approx \\ \;\;\approx M_{f}S_{s}r_{s}\left(x,p\right), \end{array}$$

where Eqs. (9) and (10) were used.

It is interesting to note that the matrix *M_f _*is not unique, but that in fact any matrix $\hat{M}=NM_{f}$ can be used for lumping, where *N *∈ ℝ^*a*×*a *^is non-singular. This observation allows us to choose a matrix $\hat{M}$ for which a maximal number of rows in $\hat{M}\;S_{s}$ vanish, which results in the greatest possible reduction in the number of state variables. Finally note that Eq. (4), in the absence of inputs, gives

(13) $$\begin{array}{lll} f_{f}\left(x,p,u_{t},t\right) & =f_{f}\left(x,p,0,t\right)=\; & \text{(1)}\\ & =S_{s}r_{s}\left(x,p\right)+S_{f}r_{f}\left(x_{f},p\right)\; & \text{(2)}\\ & \approx S_{f}r_{f}\left(x_{f},p\right)\approx 0\; & \text{(3)} \end{array}$$

since the term *S_f_r_f _*(*x_f_*, *p*) dominates the term *S_s_r_s_*(*x*, *p*). Note that Eq. (4) and consequently Eq. (13) only hold in *T*_0 _≤ *t *<*T*_1_, since the state is only known to be fast in this time span.

Eq. (12) defines the ODEs of the reduced model (the lumped state variables), and Eqs. (11) and (13) can in principle be used to calculate back-translation formulae, as is demonstrated with the small example model in the next section. However, as we shall see, this approach requires the explicit algebraic solution to a system of nonlinear equations, which is typically an infeasible task. Furthermore, there is not a clear one-to-one mapping between the state variables of the original and reduced models as in the case of proper lumping [27].

### A Small Example Model

We will now present a small example model, with three fast state variables, which is reduced with the approach discussed above. An alternative approach is then demonstrated with the advantage that it scales better to larger models.

Consider the reversible formation of a complex *C *from a substrate *A *and an enzyme *B*

$$∙\underset{}{↔}A+B\underset{k_{1}}{\overset{k_{-1}}{↔}}C\underset{}{↔}∙$$

consisting of the fast state variables *x_f _*= (*A B C*)*^T^*, where the bullets (•) represent the slow state variables surrounding the three fast state variables in the model. The ODEs for the fast state variables take the form

(14) $${ẋ}_{f}=\left(\begin{array}{c} -1\\ -1\\ 1 \end{array}\right)\left(k_{1}AB-{k_{-}}_{1}C\right)+S_{s}r_{s}\left(x,p\right).$$

The three state variables in the model constitute a fast cluster with two apparent conserved moieties, which may be represented by the following relations

(15) $$l=\left(\begin{array}{c} L_{1}\\ L_{2} \end{array}\right)=\left(\begin{array}{ccc} 1 & 0 & 1\\ 0 & 1 & 1 \end{array}\right)\left(\begin{array}{c} A\\ B\\ C \end{array}\right)=M_{f}x_{f},$$

where the lumped state variables *L*_1 _and *L*_2 _are introduced. Note that Eq. (12) defines the dynamics of the lumped state variables.

The distribution of mass among the fast state variables is given by Eq. (15) and by applying Eq. (13) to (14), which results in an equation system with the three fast state variables as unknowns

(16) $$k_{1}AB-{k_{-}}_{1}C\approx 0,$$

(17) $$A\;\text{ + }\;C\text{ = }L_{\text{1}}\text{,}$$

(18) $$B\;\text{ + }\;C\text{ = }L_{2}\text{.}$$

Analytic expressions for the fast state variables *A*, *B*, and *C *are given by the non-negative solution to Eqs. (16)-(18)

(19) $$A\approx \frac{1}{2}\left(L_{1}-L_{2}-K_{1}+\sqrt{{\left(L_{1}+L_{2}+K_{1}\right)}^{2}-4L_{1}L_{2}}\right),$$

(20) $$B\approx \frac{1}{2}\left(-L_{1}+L_{2}-K_{1}+\sqrt{{\left(L_{1}+L_{2}+K_{1}\right)}^{2}-4L_{1}L_{2}}\right),$$

(21) $$C\approx \frac{1}{2}\left(L_{1}+L_{2}+K_{1}+\sqrt{{\left(L_{1}+L_{2}+K_{1}\right)}^{2}-4L_{1}L_{2}}\right),$$

where $K_{1}=\frac{k_{-1}}{k_{1}}$.

We can now employ Eq. (12) to solve the ODEs for the lumped state variables *L*_1 _and *L*_2_, and use Eqs. (19)-(21) as back-translation formulae to compute the trajectories of the original state variables *A*, *B*, and *C*. However, note that for even slightly larger clusters of fast species than the one discussed here it would not be possible to calculate algebraic expressions of the original state variables with this approach, since it builds on the explicit solution of a system of nonlinear equations, which quickly becomes infeasible with growing problem size.

Alternatively, we can take an approach to the problem that is inspired by the method for linear systems in [27]. The first step is to express Eqs. (16) and (17) as a linear system w.r.t. the state variables *A *and *C*

(22) $$\left(\begin{array}{cc} k_{1}B & -k_{-1}\\ 1 & 1 \end{array}\right)\;\left(\begin{array}{c} A\\ C \end{array}\right)\approx \left(\begin{array}{c} 0\\ L_{1} \end{array}\right).$$

The solution to Eq. (22) w.r.t. *A *and *C *is

(23) $$A\equiv \eta_{A}\left(B,p\right)L_{1}\approx \frac{K_{1}}{B+K_{1}}L_{1},$$

(24) $$C\equiv \eta_{C}\left(B,p\right)L_{1}\approx \frac{B}{B+K_{1}}L_{1},$$

where $K_{1}=\frac{k_{-1}}{k_{1}}$, and the fraction parameters *η_A_*(*B*, *p*) and *η_C _*(*B*, *p*) are defined in Eqs. (23) and (24), respectively. The ODE for *L*_1 _is defined by Eq. (12), and the ODE for *B *can be derived by differentiation of *L*_2 _in Eq. (18), which gives that

(25) $$\begin{array}{llll} \frac{dB}{dt} & \approx {\left(1+\frac{K_{1}L_{1}}{{\left(B+K_{1}\right)}^{2}}\right)}^{-1}\left(\begin{array}{cc} -\frac{B}{B+K_{1}} & 1 \end{array}\right)\left(\begin{array}{c} \frac{L_{1}}{dt}\\ \frac{L_{2}}{dt} \end{array}\right)=\; & \text{(1)}\\ & ={\left(1+\frac{K_{1}L_{1}}{{\left(B+K_{1}\right)}^{2}}\right)}^{-1}\left(\begin{array}{cc} -\frac{B}{B+K_{1}} & 1 \end{array}\right)\cdot \; & & \;\\ & \cdot M_{f}S_{s}r_{s}\left(x,k\right),\; & & \; \end{array}$$

The reduced model consists of the two state variables *L*_1 _and *B *(note that *L*_2 _does not appear in the reduced model), and the dynamics is described by Eqs. (12) and (25), respectively. Note that the state variables *A *and *C *can be back-translated from the reduced model with Eqs. (23) and (24). This approach is a bit more intricate than the first, but comes with the advantage that we do not need to solve a system of nonlinear equations.

### A Method for Zooming of Nonlinear Models

We will now step-by-step present a method that can be used to construct zoomable nonlinear biochemical models. This involves two sub-goals: i) to identify a reduced model that shares important characteristics with the original model, ii) to derive back-translation formulae that can be used to compute the original state variables and parameters from the reduced model.

In an initialization step of the method for a model ${ℳ}_{o}$ we first formulate mathematical equations for all conservation relations Eq. (8), state variables in steady-state Eq. (3), and quasi-steady state assumptions Eq. (4). If additional properties of the system are known, we also formulate the corresponding equations.

### Step 1

The first step of the method is to identify the apparent conservation relations in the model.

**Definition 1: **Let *S_f _*be the stoichiometric matrix for the reactions *r_f _*(*x_f_*, *p*) as defined in Eq. (9). Each subset of state variables for which the corresponding rows of *S_f _*are linearly dependent constitutes an apparent conservation relation. Hence the apparent conservation relations lie in the left null space of *S_f _*and the dimension of this space is *n *- rank(*S_f_*).

Note that the apparent conservation relations are defined in Eq. (11). It is trivial to identify the set of all linearly dependent rows of *S_f _*with a mathematical computing software (e.g., SBtoolbox for Matlab [36]).

### Step 2

The second step of the method is to define the state variables of the reduced model, which we refer to as modified lumped state variables.

**Definition 2: **Let *x *be a lumped state variable corresponding to a subset of the state variables in an apparent conservation relation. Then *x *is a modified lumped state variable if the lumping scheme with respect to the state variables of the original model is proper.

Note that the original state variables have a clear interpretation in the reduced model (i.e., that the lumped variables form disjoint sets) if the lumping scheme is proper, i.e.,

(26) $$l_{m}=M_{m}x_{f}$$

where *M_m _*is *a *× *n_f _*matrix with elements equal to 0 or 1 and column sums equal to 1, and *l_m _*denotes the modified lumped state variables. We typically have a large freedom in the choice of *M_m_*. The number of state variables is maximally reduced if all exact conservation relations in the model are retained as modified lumped state variables (and replaced by constants).

### Step 3

The third step of the method is to derive fraction parameters, which constitute the link between the reduced model and the original model. Let the original state variables that constitute the *k*:th modified lumped state variable $l_{m_{k}}$ be denoted by $x_{m_{k}}$, so that

(27) $$l_{m_{k}}=\overset{w}{\underset{i=1}{\sum }}x_{m_{ki}},$$

A number of *n_m _*equations that are linear w.r.t. $x_{m_{k}}$, and linearly independent, are required to calculate fraction parameters. The existance of *n_m _*such equations results in an equation system

(28) $$b_{k}\text{(}l_{m}\text{,}p\text{)}\;\text{ = }\;A\text{(}l_{m}\text{,}p\text{)}x_{m_{k}},$$

where both $A\left(l_{m},p\right)\in {{\text{R}}^{n_{m}}}^{\times n_{m}}$ and $b_{k}\left(l_{m},p\right)\in {\text{R}}^{n_{m}}$ are known, although some of the equations may in general be approximate (e.g., QSS). The matrix *A*(*l_m_*, *p*) is invertible since the equations are linearly independent, and we have that

(29) $$x_{m_{k}}=A^{-1}\left(l_{m},p\right)b_{k}\left(l_{m},p\right).$$

The fraction parameters can then be calculated

(30) $$\eta_{m_{ki}}\left(l_{m},p\right)=\frac{x_{m_{ki}}}{l_{m_{k}}}=\frac{x_{m_{ki}}}{\sum_{i=1}^{n_{m}}x_{m_{ki}}},$$

where we used Eq. (27) in the last step.

A modified lumped state variable for which an insufficient number of linear and linearly independent equations are available may still be used in the reduced model. However, the back-translation of the modified lumped state variable to the original state variables is then not possible, and step 3 of the method is ignored.

### Step 4

The fourth step of the method is to derive the rate of change of the modified lumped state variables. **Theorem: **The dynamics of the modified lumped state variables is given by

(31) $$\begin{array}{lll} {\overset{{}^{\circ}}{l}}_{m} & ={\left(I+J\left(l_{m},p\right)\right)}^{-1}\overset{{}^{\circ}}{l}=\; & \text{(1)}\\ & ={\left(I+J\left(l_{m},p\right)\right)}^{-1}M_{f}S_{s}r_{s}\left(x,p\right),\; & \text{(2)} \end{array}$$

where *S_s _*and *r_s_*(*x*, *p*) were defined in Eq. (9), the matrix *M_f _*is defined in Eq. (11), and

(32) $$J_{ij}\left(l_{m},p\right)=\underset{k}{\sum }\left(M_{f_{ik}}-M_{m_{ik}}\right)\frac{\partial gk\left(l_{m},p\right)}{\partial l_{m_{j}}},$$

where the matrix *M_m _*is defined in Eq. (26), and $g_{i}\left(l_{m},p\right)\equiv x_{f_{i}}$ is introduced to simplify the notation.

Proof:

First subtract Eq. (26) from Eq. (11)

$$l\;=l_{m}+\left(M_{f}-M_{m}\right)x_{f}=l_{m}+\left(M_{f}-M_{m}\right)g\left(l_{m},p\right),$$

and differentiate *l *with respect to time, which gives

(33) $$\overset{{}^{\circ}}{l}\text{ = }{\overset{{}^{\circ}}{l}}_{m}\text{ + }J\text{(}l_{m}\text{,p)}{\overset{{}^{\circ}}{l}}_{m}\text{ = (}I\text{ + }\;J\text{(}l_{m}\text{,}p\text{))}{\overset{{}^{\circ}}{l}}_{m}\text{,}$$

where *I *is the identity matrix and *J*(*l_m_*, *p*) is the Jacobian of (*M_f _*- *M_m_*)*g*(*l_m_*, *p*) with respect to *l_m_*. The element *J_ij _*of *J*(*l_m_*, *p*) is given by

$$J_{ij}\left(l_{m},p\right)=\underset{k}{\sum }\left(M_{f_{ik}}-M_{m_{ik}}\right)\frac{\partial g_{k}\left(l_{m},p\right)}{\partial l_{m_{j}}}.$$

From Eq. (33) it is straight-forward to derive ${\overset{{}^{\circ}}{l}}_{m}$, which takes the form

$$\begin{array}{lll} {\overset{{}^{\circ}}{l}}_{m} & ={\left(I+J\left(l_{m},p\right)\right)}^{-1}\overset{{}^{\circ}}{l}=\; & \text{(1)}\\ & ={\left(I+J\left(l_{m},p\right)\right)}^{-1}M_{f}S_{s}r_{s}\left(x,p\right),\; & \text{(2)} \end{array}$$

where Eq. (12) was used in the last step. □

The matrix *I *+ *J*(*l_m_*; *p*) is symbolically invertible, but may in general contain singularities for particular combinations of parameters values and state variable values. However, the matrix is always invertible for the models discussed in this paper, since the corresponding determinants are strictly positive.

### Step 5

The final step of the method is to back-translate the modified lumped state variables to the original state variables with the fraction parameters derived in step 3. This allows a comparison between the predictions by the reduced model to those of the original model.

The implementation of the method is straight-forward, and we have used Matlab (R2008b) together with the SBtoolbox [36] as computing software for the models in this paper.

## Results

We will now demonstrate the method through application to two example models.

### Enzyme Kinetics Model

The model below describes the process of conversion of a substrate, *S*, into a product, *P*, which is catalyzed by an enzyme, *E*.

$$S+E\underset{k_{1}}{\overset{k_{-1}}{↔}}C_{S}\overset{}{\underset{k_{2}}{\to }}C_{P}\underset{k_{3}}{\overset{k_{-3}}{↔}}P+E$$

Note that the complexes *C_s _*and *C_p _*are formed by *S *bound to *E*, and *P *bound to *E*, respectively. This model is frequently occurring as part of larger models of biological systems, although the reaction from *C_S _*to *C_P _*is sometimes neglected, or reversible. The ODEs for the model are listed in Appendix A.1, where the three reactions are defined as: *r*_1 _= *k*_1_*SE *- *k*_-1_*C_S_*, *r*_2 _= *k*_2_*C_S_*, and *r*_3 _= *k*_3_*C_P _*- *k*_-3_*PE*.

The reaction terms *r*_1_(*x*, *p*) and *r*_3_(*x*, *p*) are assumed to be dominating, and the reaction term *r*_2_(*x*, *p*) to be insignificant in the ODEs. This results in that all state variables are in QSS, which gives

(34) $$k_{1}SE-{k_{-}}_{1}C_{S}\approx 0,$$

(35) $$k_{3}PE-k_{-3}C_{P}\approx 0.$$

We denote the sum of the state variables containing the enzyme by

(36) $$L_{E}=E+C_{S}+C_{P}\text{,}$$

which is constant since the total amount of the enzyme *E *is conserved in the system.

#### Reduction of the Enzyme Kinetics Model

The first step of the method is to identify the apparent conservation relations from the matrix *S_f_*. Since *r*_2_(*x*, *p*) is dominated by *r*_1_(*x*, *p*) and *r*_3_(*x*, *p*) the model ODEs can be written on the form of Eq. (9)

$$\begin{array}{llll} \left(\begin{array}{c} Ṡ\\ Ė\\ Ṗ\\ \overset{{}^{\circ}}{C_{S}}\\ \overset{{}^{\circ}}{C_{P}} \end{array}\right) & =S_{s}r_{s}\left(x,p\right)+S_{f}r_{f}\left(x_{f},p\right)=\; & & \;\\ & =\left(\begin{array}{c} 0\\ 0\\ 0\\ -1\\ 1 \end{array}\right)r_{2}+\left(\begin{array}{cc} -1 & 0\\ -1 & 1\\ 0 & 1\\ 1 & 0\\ 0 & -1 \end{array}\right)\left(\begin{array}{c} r_{1}\\ r_{3} \end{array}\right),\; & & \; \end{array}$$

A basis of the left null space of *S_f _*is given by the row vectors of *M_f_*, which is defined by

(37) $$\begin{array}{lll} l & =\left(\begin{array}{c} L_{S}\\ L_{P}\\ L_{E} \end{array}\right)=\; & \text{(1)}\\ & =\left(\begin{array}{ccccc} 1 & 0 & 0 & 1 & 0\\ 0 & 0 & 1 & 0 & 1\\ 0 & 1 & 0 & 1 & 1 \end{array}\right)\left(\begin{array}{c} S\\ E\\ P\\ C_{S}\\ C_{P} \end{array}\right)=\; & \text{(2)}\\ & =M_{f}x_{f},\; & \text{(3)} \end{array}$$

where *L_S _*and *L_P _*are apparent conservation relations and *LE *is an exact conservation relation.

The second step is to define the modified lumped state variables on the form of Eq. (26). The number of state variables is maximally reduced if *L_E _*is retained as a state variable in the reduced model (i.e., since *L_E_*, unlike *L_S _*and *L_P_*, can be replaced by a constant). The vector of modified lumped state variables then is defined

$$\begin{array}{llll} l_{m} & =\left(\begin{array}{c} S\\ P\\ L_{E} \end{array}\right)=\left(\begin{array}{ccccc} 1 & 0 & 0 & 0 & 0\\ 0 & 0 & 1 & 0 & 0\\ 0 & 1 & 0 & 1 & 1 \end{array}\right)\left(\begin{array}{c} S\\ E\\ P\\ C_{S}\\ C_{P} \end{array}\right)=\; & & \;\\ & =M_{f}x_{f},\; & & \; \end{array}$$

In the third step of the method we calculate fraction parameters for the modified lumped state variable *L_E_*. There are five equations (Eqs. (34)-(35) and (37)) that are linear w.r.t. the state variables *E*, *C_S_*, and *C_P_*, which are lumped into the state variable *L_E_*. Note that only *n_m _*= 3 equations are required to derive fraction parameters, and we use Eqs. (34)-(36) to formulate an equation system as in Eq. (28) with the solution

(38) $$E=\eta_{E}L_{E}\approx \frac{1}{1+M_{1}S+M_{3}P}L_{E}\equiv g_{2}\left(l_{m},p\right),$$

(39) $$C_{S}=\eta_{C_{S}}L_{E}\approx \frac{M_{1}S}{1+M_{1}S+M_{3}P}L_{E}\equiv g_{4}\left(l_{m},p\right),$$

(40) $$C_{P}=\eta_{C_{P}}L_{E}\approx \frac{M_{3}P}{1+M_{1}S+M_{3}P}L_{E}\equiv g_{5}\left(l_{m},p\right),$$

where $M_{1}=\frac{k_{1}}{k-1}$ and $M_{3}=\frac{k_{3}}{k-3}$. The two remaining modified lumped state variables correspond to *S *and *P *in the original model, so we define that $l_{m_{1}}=S\equiv g_{1}\left(l_{m},p\right)$ and $l_{m_{2}}=P\equiv g_{3}\left(l_{m},p\right)$.

In the fourth step we derive the rate of change of the modified lumped state variables. Eq. (32) gives that ${\overset{{}^{\circ}}{L}}_{E}=0$, which is replaced by a constant, and

(41) $$Ṡ\approx \frac{-k_{2}M_{1}SL_{E}\left({\left(1+M_{1}S+M_{3}P\right)}^{2}+M_{3}L_{E}\right)}{\phi \left(S,P,L_{E},M_{1},M_{3}\right)},$$

(42) $$Ṗ\approx \frac{k_{2}M_{1}SL_{E}\left({\left(1+M_{1}S+M_{3}P\right)}^{2}+M_{1}L_{E}\right)}{\phi \left(S,P,L_{E},M_{1},M_{3}\right)}.$$

where

$$\phi \left(S,P,L_{E},M_{1},M_{3}\right)=\left({\left(1+M_{1}S+M_{3}P\right)}^{3}+\left(M_{1}+M_{3}+M_{1}M_{3}\left(P+S\right)\right)\left(1+M_{1}S+M_{3}P\right)L_{E}+M_{1}M_{3}L_{E}^{2}\right).$$

The two ODEs in Eqs (41)-(42) define the dynamics of the state variables in the reduced model. We finally note that the exact conservation relation for the substrate, *L_T _*= *S *+ *P *+ *C_S _*+ *C_P_*, together with Eqs. (39)-(40) can be used to reduce the model further to a single state.

**Figure 1:**
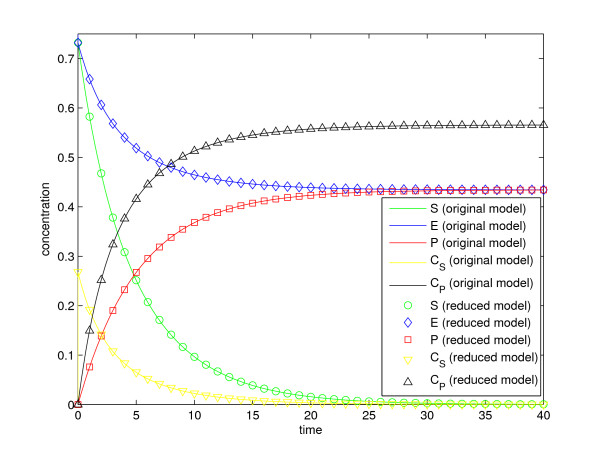
**Small example model**. A comparison between the state variables of the original enzyme kinetics model and the backtranslated state variables of the reduced version of the same model.

In the fifth step of the method we use the fraction parameters, defined in Eqs. (38)-(40), to back-translate the modified lumped state variables to the state variables of the original model. A comparison between predictions of the original state variables, from simulations of the original model and the reduced model, is presented in Figure 1. Implementations of the original model (Additional file 1), the reduced model (Additional file 2), and a script for simulation with SBtoolbox2 for MATLAB [36] (Additional file 3), are available in *Additional files*.

The only assumption that was used in the derivation of the reduced model is that the reaction terms *r*_1_(*x*, *p*) and *r*_3_(*x*, *p*) dominate the reaction term *r*_2_(*x*, *p*), which results in that all state variables are in QSS. To assess the impact of these assumptions on the reduced model we compute the relative difference between the state variables in the original and in the reduced model

$$\varepsilon_{i}\left(t\right)=\frac{\left|x_{i}^{o}\left(t\right)-x_{i}^{r}\left(t\right)\right|}{x_{i}^{o}\left(t\right)},\;i=1,\;\ldots ,\;n,$$

where $x_{i}^{o}$ is state variable *i *in the original model, $x_{i}^{r}$ is the corresponding back-translated state variable in the reduced model, and |*x*| denotes the elementwise absolute values of *x*. The maximal mean and infinity norm of *ε_i_*(*t*) in Eq. (43) over time is presented in Table 1 for parameter values over five orders of magnitude. In general, the reduced model appears to be robust to changes in the parameter values, although slightly more sensitive to some parameters (e.g., small values of *k*_-1_, large values of *k*_1_, or large values of *k*_2_, which violate the assumptions used in the reduction). However, note that the validity of the QSS assumption may also depend on the state variables, for example the total concentration of the enzyme. Interestingly, we observed that the reduced model can well approximate the original model over several order of magnitudes around the nominal enzyme concentration (*L_E _*= 1). It is well-known that the QSS approximation is only valid for sufficiently small enzyme concentrations, and as expected the performance of the reduced model starts to decrease for immense enzyme concentrations.

**Table 1:** Robustness of the reduced model for large deviations from the nominal parameter point are presented for the enzyme kinetics model, with a sampling frequency of 0.1 (starting from 0.1) time units.

Param./Factor	10^-2^	10^-1^	10^0^	10^1^	10^2^
*k*_1_	0.00053/0.0082	0.0018/0.0071	0.0010/0.0059	0.019/0.18	0.19/1.8
*k*_-1_	0.19/1.9	0.0061/0.079	0.0010/0.0059	0.0017/0.0025	0.00024/0.0028
*K*_2_	0.00019/0.0060	0.00082/0.0055	0.0010/0.0059	0.035/0.23	0.21/0.39
*k*_3_	0.0068/0.014	0.0053/0.010	0.0010/0.0059	0.0067/0.044	0.035/0.29
*K*_-3_	0.020/0.30	0.0048/0.032	0.0010/0.0059	0.0051/0.0093	0.0069/0.015
All	0.012/1.2	0.0014/0.062	0.0010/0.0059	0.025/0.41	0.010/0.045

The nominal parameter values (*k*_1 _= 1000, *k*_-1 _= 2000, *k*_2 _= 1, *k*_3 _= 1000, and *k*_-3 _= 3000) are modified by a multiplicative factor, and the maximal (for any state variable) time average/infinity norm of the relative difference between the original and the reduced model is presented above. Note that only concentrations larger than 10^-6 ^are considered in the analysis above, due to potential numerical inaccuracies.

Note that all the state variables of the original model have a direct biological interpretation also in the reduced model, and that Eqs. (38)-(40) can be used to back-translate the state variables. The reduced model may be depicted

$$S\;+\eta_{E}L_{E}\underset{k_{1}}{\overset{k_{-1}}{↔}}\eta_{C_{S}}L_{E}\overset{}{\underset{k_{2}}{\to }}\eta_{C_{P}}L_{E}\underset{k_{3}}{\overset{k_{-3}}{↔}}P+\eta_{E}L_{E}$$

where the fraction parameters specify the distribution of the enzyme among the corresponding original state variables.

### Glucose Transport in Budding Yeast

**Figure 2:**
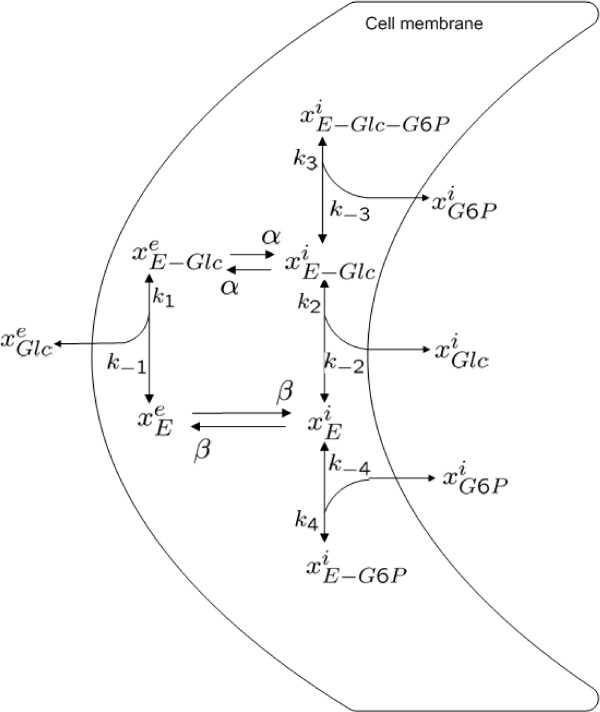
**Glucose transport model**. The original model for glucose transport in baker's yeast (*S. cerevisiae*). This figure was originally presented in [27].

A model for the transport of glucose into a cell of baker's yeast (*S. cerevisiae*), which constitutes the first step of glycolysis, is presented in [37]. The inflow of glucose is modeled as a facilitated diffusion process, in which a carrier enzyme is responsible for the transport between the inner and outer regions of the cellular membrane. It is assumed that glucose 6-phosphate (G6P) has an inhibitory role in the glucose transport process by binding to the transporter. A graphical representation of the model is shown in Figure 2, and the ODEs for the state variables are listed in Appendix A.2.

In [27] we described how the calculation of fraction parameters, based on a set of assumptions, leads to the same reaction rates in the reduced model as were reported in [37]. The assumptions are that state variables participating in reactions for uptake and release of glucose and G6P across the cell membrane are in QSS, that the transporter is conserved, and that the concentrations of the transporter in the inner and outer regions of the cellular membrane are constant.

The assumption that the state variables $x_{Glc}^{e},\;x_{Glc}^{i},\;x_{E-G6P}^{i}$, and $x_{E-Glc-G6P}^{i}$, which participate in the uptake and release of G6P and glucose across the cell membrane, are in QSS gives that

(43) $$k_{1}x_{E}^{e}x_{Glc}^{e}-k_{-1}x_{E-Glc}^{e}\approx 0,$$

(44) $$k_{2}x_{E}^{i}x_{Glc}^{i}-k_{-2}x_{E-Glc}^{i}\approx 0,$$

(45) $$k_{4}x_{E}^{i}x_{G6P}^{i}-k_{-4}x_{E-G6P}^{i}\approx 0,$$

(46) $$k_{3}x_{E-Glc}^{i}x_{G6P}^{i}-k_{-3}x_{E-Glc-G6P}^{i}\approx 0.$$

We have the following exact conservation relations in the model

(47) $$\begin{array}{lll} L_{E}= & x_{E-G6P}^{i}+x_{E-Glc-G6P}^{i}+x_{E-Glc}^{e}+\ldots \; & \text{(1)}\\ & x_{E-Glc}^{i}+x_{E}^{e}+x_{E}^{i},\; & \text{(2)}\\ & \; & \text{(3)} \end{array}$$

(48) $$L_{Glc}=x_{Glc}^{e}+x_{Glc}^{i}+x_{E-Glc-G6P}^{i}+x_{E-Glc}^{e}+x_{E-Glc}^{i},$$

(49) $$L_{G6P}=x_{G6P}^{i}+x_{E-G6P}^{i}+x_{E-Glc-G6P}^{i},$$

where *L_E_*, *L_Glc_*, and *L*_*G*6*P *_are constant over time. The assumption in [37] that the concentrations of the transporter in the inner and outer regions of the cell membrane are constant is formulated

(50) $$\alpha \left(x_{E-Glc}^{e}-x_{E-Glc}^{i}\right)+\beta \left(x_{E}^{e}-x_{E}^{i}\right)=0.$$

It is not clear how equations for back-translation of the state variables in the reduced model in [27,37] can be derived. The reduced model has three state variables; external- and internal glucose and G6P, but only two differential equations for the in- and outflow of glucose, since the ODE for G6P is replaced by a representative function that is inferred from the G6P data. Our method does not rely on that such information is available, although it would in principle be possible to utilize data fitted functions for the state variables. Also note that the equations in the reduced model describe the total influx and efflux of glucose across the membrane [27], which cannot be interpreted w.r.t. the state variables of the original model. Other assumptions in [37] that complicates a comparison with our method are that the efflux of glucose is negligible, that the concentration of glucose in the cytosol is negligible, and that the concentrations of the transporter are constant in the inner and outer regions of the cell membrane (Eq. (50)).

It is not possible to generate a reduced model by direct substitution of the fraction parameters that were derived in [27] into the ODEs of the original model, since this would lead to the prediction that the state variables are constant (as discussed in [27]). We will now instead illustrate how our method can be used to derive a reduced and zoomable version of the glucose transport model.

#### Reduction of the Glucose Transport Model

Before applying our method to the glucose transport model we tried an alternative approach. Eqs. (43)-(46) were solved w.r.t. the state variables in QSS, and the resulting expressions were then substituted into the remaining ODEs. The details of the derivation of the reduced model are presented in Appendix A.3. The reduced model does not produce satisfactory predictions for any other state variable than $x_{G6P}^{i}$, which remains approximately constant during the simulation. Implementations of the original model (Additional file 4), the reduced model (Additional file 5), and a script for simulation with SBtoolbox2 for MATLAB [36] (Additional file 6), are available in *Additional files*.

Since the first approach turned out to be insufficient for reduction of the glucose transport model we applied our method to the same model. Following [37], we initially assumed constant transporter concentrations in the inner and outer regions of the cellular membrane, as defined by Eq. (50). For the details on the derivation of the reduced model we refer to Appendix A.4. The reduced model clearly performs better than the model resulting from the first approach, but it is still not satisfactory. However, the assumption of constant regional concentrations of the transporter may not be valid since the transport of glucose across the cell membrane is a rate limiting step in the model, and appears to be important for the state variable dynamics. We therefore decided to neglect Eq. (50) in the reduction process.

Implementations of the original model (Additional file 4), the reduced model (Additional file 7), and a script for simulation with SBtoolbox2 for MATLAB [36] (Additional file 8), are available in *Additional files*. In the first step of the method we identify the following apparent conservation relations

(51) $$\begin{array}{llll} l= & \left(\begin{array}{c} L_{Glc_{1}}\\ L_{E_{1}}\\ L_{Glc_{2}}\\ L_{G6P}\\ L_{E_{2}} \end{array}\right)=Mx=\; & \text{(1)}\\ = & \left(\begin{array}{ccccccccc} 1 & 0 & 0 & 0 & 0 & 1 & 0 & 0 & 0\\ 0 & 0 & 0 & 0 & 0 & 1 & 0 & 1 & 0\\ 0 & 1 & 0 & 1 & 0 & 0 & 1 & 0 & 0\\ 0 & 0 & 1 & 1 & 1 & 0 & 0 & 0 & 0\\ 0 & 0 & 1 & 1 & 0 & 0 & 1 & 0 & 1 \end{array}\right)x.\; & & \; \end{array}$$

We note that there are two disjoint clusters of fast reactions in the model, corresponding to the outer- and inner parts of the cell membrane.

In the second step we define the modified lumped state variables. We decide to keep the lumped state variables $L_{E_{1}}$ and *L*_*G*6*P *_as modified lumped state variables. The choice to keep *L*_*G*6*P *_leads to the largest possible reduction in the number of state variables, since the conservation of *L*_*G*6*P *_is exact (which is not true for any other state variable in *l*). The modified lumped state variables are defined true for any other state variable in *l*). The modified lumped state variables are defined

(52) $$\begin{array}{llll} l_{m}= & \left(\begin{array}{c} x_{Glc}^{e}\\ L_{E_{1}}\\ x_{Glc}^{i}\\ L_{G6P}\\ L_{E_{3}} \end{array}\right)=M_{m}x=\; & \text{(1)}\\ = & \left(\begin{array}{ccccccccc} 1 & 0 & 0 & 0 & 0 & 0 & 0 & 0 & 0\\ 0 & 0 & 0 & 0 & 0 & 1 & 0 & 1 & 0\\ 0 & 1 & 0 & 0 & 0 & 0 & 0 & 0 & 0\\ 0 & 0 & 1 & 1 & 1 & 0 & 0 & 0 & 0\\ 0 & 0 & 0 & 0 & 0 & 0 & 1 & 0 & 1 \end{array}\right)x,\; & & \; \end{array}$$

where

$$x=\;\left(\begin{array}{c} x_{Glc}^{e}\\ x_{Glc}^{i}\\ x_{E-G6P}^{i}\\ x_{E-Glc-G6P}^{i}\\ x_{G6P}^{i}\\ x_{E-Glc}^{e}\\ x_{E-Glc}^{i}\\ x_{E}^{e}\\ x_{E}^{i} \end{array}\right).$$

Note that two of the state variables in the original model, $x_{Glc}^{e}$ and $x_{Glc}^{i}$, are also modified lumped state variables.

In the third step of the method we calculate fraction parameters for the modified lumped state variables that correspond to more than one of the original state variables (i.e., $L_{E_{1}}$, *L*_*G*6*P*_, and $L_{E_{3}}$). All of the modified lumped state variables satisfy the requirement that at least *n_m _*of Eqs. (43)-(49) and Eq. (52) are linear, and linearly independent, with respect to the corresponding original state variables. Eqs. (43) and (52) form a nonlinear equation system with the solution

(53) $$\begin{array}{llllll} \left(\begin{array}{c} x_{E}^{e}\\ x_{E-Glc}^{e} \end{array}\right)= & \left(\begin{array}{c} \eta_{E}^{e}\\ \eta_{E-Glc}^{e} \end{array}\right)L_{E_{1}}\approx \; & & \; & \text{(1)}\\ \approx & \frac{1}{\left(K_{1}+x_{Glc}^{e}\right)}\left(\begin{array}{c} K_{1}\\ x_{Glc}^{e} \end{array}\right)L_{E_{1}}.\; & & \; & & \; \end{array}$$

Let us define that $g_{6}\equiv x_{E-Glc}^{e}$ and $g_{8}\equiv x_{E}^{e}$. Similarly, the fractions of the two carrier state variables in the inner regions of the cell to the lumped state variable $L_{E_{3}}$ can be computed from Eqs. (44) and (52) (Eq. (28))

(54) $$\begin{array}{llll} \left(\begin{array}{c} x_{E-Glc}^{i}\\ x_{E}^{i} \end{array}\right)= & \left(\begin{array}{c} \eta_{E-Glc}^{i}\\ \eta_{E}^{i} \end{array}\right)L_{E_{3}}\approx \; & \text{(1)}\\ & \approx \frac{1}{\left(x_{Glc}^{i}+K_{2}\right)}\left(\begin{array}{c} x_{Glc}^{i}\\ K_{2} \end{array}\right)L_{E_{3}}.\; & & \; \end{array}$$

We define that $g_{7}\equiv x_{E-Glc}^{i}$ and $g_{9}\equiv x_{E}^{i}$. The fraction parameters for the G6P-state variables can be computed from Eqs. (45)-(46) and Eq. (28) with the solution

(55) $$\begin{array}{llll} & \left(\begin{array}{c} x_{E-G6P}^{i}\\ x_{E-Glc-G6P}^{i}\\ x_{G6P}^{i} \end{array}\right)=\left(\begin{array}{c} \eta_{E-G6P}^{i}\\ \eta_{E-Glc-G6P}^{i}\\ \eta_{G6P}^{i} \end{array}\right)L_{G6P}\approx \; & \text{(1)}\\ & \approx \frac{1}{\xi }\left(\begin{array}{c} K_{3}x_{E}^{i}\\ K_{4}x_{E-Glc}^{i}\\ K_{3}K_{4} \end{array}\right)L_{G6P}.\; & & \; \end{array}$$

where $\xi =K_{3}K_{4}+K_{4}x_{E-Glc}^{i}+K_{3}x_{E}^{i}$. We define that $g_{3}\equiv x_{E-G6P}^{i}$, $g_{4}\equiv x_{E-Glc-G6P}^{i}$, and $g_{5}\equiv x_{G6P}^{i}$. For the two original state variables that are kept as modified lumped state variables in the reduced model we define that $g_{1}\equiv x_{Glc}^{e}$ and $g_{2}\equiv x_{Glc}^{i}$.

The fourth step of the method is to derive rate equations for the modified lumped state variables. Since the apparent conservations are separated into two disjoint clusters of fast state variables, we can treat the model for the inner and outer regions of the membrane separately. Let the modified lumped state variables corresponding to the outer region of the cell membrane be denoted by $l_{m_{1}}={\left(x_{Glc}^{e}\;L_{E_{1}}\right)}^{T}$, and the variables in the inner region of the cell membrane by $l_{m_{2}}={\left(x_{Glc}^{i}\;L_{E_{3}}\right)}^{T}$. Note that the state variable *L*_*G*6*P *_can be replaced by a constant in the model, since ${\overset{{}^{\circ}}{L}}_{G6P}=0$. The ODEs of the modified lumped state variables are derived with Eq. (31). In the inner region the ODEs are

(56) $$\begin{array}{llll} {\overset{{}^{\circ}}{l}}_{m_{1}}= & \left(\begin{array}{c} {ẋ}_{Glc}^{e}\\ {\overset{{}^{\circ}}{L}}_{E_{1}} \end{array}\right)={\left(I+J\right)}^{-1}{\overset{{}^{\circ}}{l}}_{1}=\; & \text{(1)}\\ & ={\left(\begin{array}{cc} 1+J_{11} & J_{12}\\ 0 & 1 \end{array}\right)}^{-1}{\overset{{}^{\circ}}{l}}_{1},\; & & \; \end{array}$$

where

(57) $$\begin{array}{llll} {\overset{{}^{\circ}}{l}}_{1}= & \left(\begin{array}{c} {\overset{{}^{\circ}}{L}}_{Glc_{1}}\\ {\overset{{}^{\circ}}{L}}_{E_{1}} \end{array}\right)\approx \; & \text{(1)}\\ & \approx \left(\begin{array}{c} -\alpha \left(x_{EGlc}^{e}-x_{EGlc}^{i}\right)\\ -\alpha \left(x_{EGlc}^{e}-x_{EGlc}^{i}\right)-\beta \left(x_{E}^{e}-x_{E}^{i}\right) \end{array}\right).\; & & \; \end{array}$$

In the larger outer region of the cell membrane the ODEs take the form

(58) $$\begin{array}{llll} {\overset{{}^{\circ}}{l}}_{m_{2}}\approx & \left(\begin{array}{c} {ẋ}_{Glc}^{i}\\ {\overset{{}^{\circ}}{L}}_{E_{3}} \end{array}\right)={\left(I+J\right)}^{-1}{\overset{{}^{\circ}}{l}}_{2}=\; & \text{(1)}\\ = & {\left(\begin{array}{cc} 1+J_{11} & J_{12}\\ J_{21} & 1+J_{22} \end{array}\right)}^{-1}{\overset{{}^{\circ}}{l}}_{2},\; & & \; \end{array}$$

where

(59) $$\begin{array}{llll} {\overset{{}^{\circ}}{l}}_{2}= & \left(\begin{array}{c} {\overset{{}^{\circ}}{L}}_{Glc_{2}}\\ {\overset{{}^{\circ}}{L}}_{E_{2}} \end{array}\right)\approx \; & \text{(1)}\\ \approx & \left(\begin{array}{c} -\alpha \left(x_{EGlc}^{i}-x_{EGlc}^{e}\right)\\ -\alpha \left(x_{EGlc}^{i}-x_{EGlc}^{e}\right)-\beta \left(x_{E}^{i}-x_{E}^{e}\right) \end{array}\right).\; & & \; \end{array}$$

**Figure 3:**
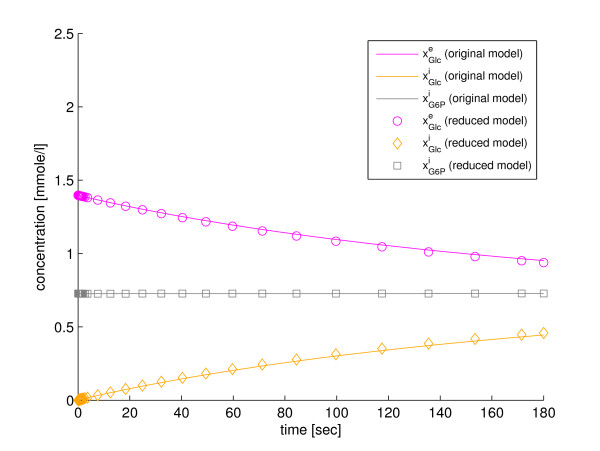
**Reduction with our method to four state variables**. A comparison between the original glucose transport model and the model reduced to four state variables with our method, w.r.t. the state variables of the original model.

**Figure 4:**
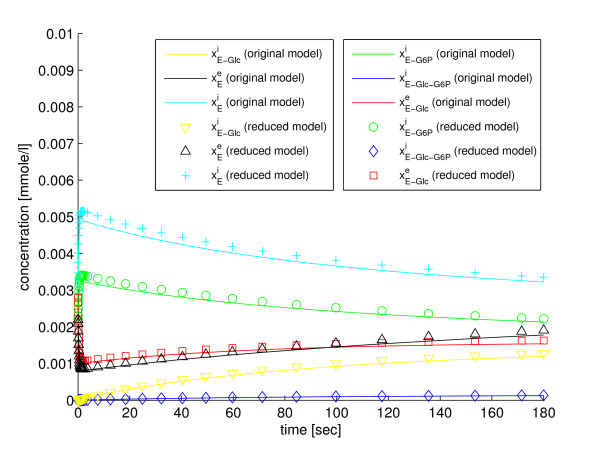
**Reduction with our method to four state variables**. A comparison between the original glucose transport model and the model reduced to four state variables with our method, w.r.t. the state variables of the original model.

In the fifth step of the method the reduced model, which is defined by the four ODEs in Eqs. (56)-(59), is simulated. The trajectories of the state variables of the reduced model can be back-translated to the original state variables with the fraction parameters defined in Eqs. (53), (54), and (55). The simulation results are shown in Figure 3 and Figure 4. All the state variables can be back-translated properly, which shows that the model properties that are important for recovery of the state variables are retained in the reduction. Implementations of the original model (Additional file 4), the reduced model (Additional file 9), and a script for simulation with SBtoolbox2 for MATLAB [36] (Additional file 10), are available in *Additional files*.

**Figure 5:**
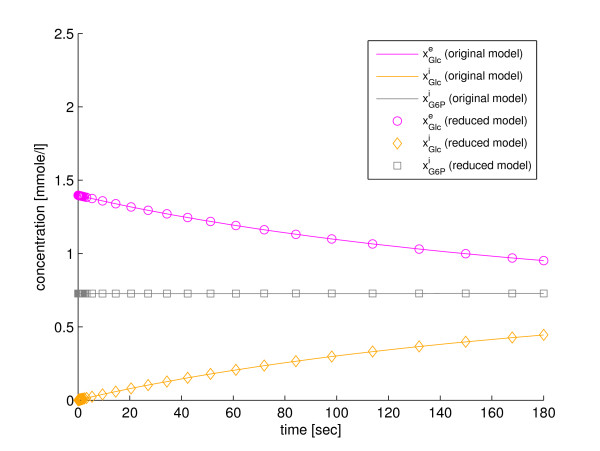
**Reduction with our method to five state variables**. A comparison between the original glucose transport model and the model reduced to five state variables with our method, w.r.t. the state variables of the original model.

**Figure 6:**
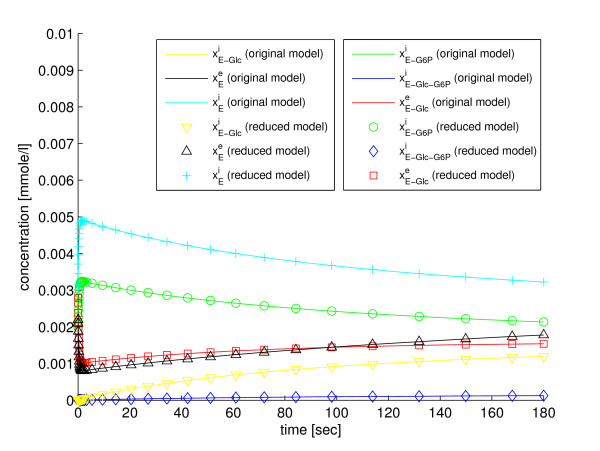
**Reduction with our method to five state variables**. A comparison between the original glucose transport model and the model reduced to five state variables with our method, w.r.t. the state variables of the original model.

If we use $L_{E_{2}}$ instead of *L*_*G*6*P *_as a modified lumped state variable, the reduced model will have the same state variables as the reduced model in [27,37] (i.e., $x_{Glc}^{e},\;x_{Glc}^{i}$, and $x_{G6P}^{i})$ two additional state variables for the transporter. This gives a reduced model with five state variables, but equally many parameters as in the previous case. A comparison between the original model and the reduced model, w.r.t. the original state variables, is shown in Figure 5 and Figure 6. As can be seen the comparison is very good, in fact it is even slightly better than for the reduced model with four state variables. The details of the derivation of the reduced model are presented in Appendix A.5. Implementations of the original model (Additional file 4), the reduced model (Additional file 11), and a script for simulation with SBtoolbox2 for MATLAB [36] (Additional file 12), are available in *Additional files*.

Note that the only assumption used to derive the reduced model is that states that are involved in reactions at the membrane are in QSS. To investigate the parameter space region in which the QSS assumptions are valid we use the measure defined in Eq. (43). The maximal mean and infinity norm of the relative difference between the original and the reduced model in Eq. (43) over time is presented in Table 2. The reduced model appears to be relatively robust to changes in the parameters, although sensitive to small values of *k*_-4 _and to large values of *k*_4_. This is mainly due to that a large proportion of the transporter *E *is absorbed in $x_{E-G6P}^{i}$, which leads to that some of the QSS assumptions are invalid. We also observed that relative difference between the models is insensitive to the total concentration of the transporter for several orders of magnitude around the nominal value (*L_E _*= 0.01). However, note that this observation is specific to the studied model and may not be generalizable to other similar biochemical models.

**Table 2:** Robustness of the reduced model for large deviations from the nominal parameter point are presented for the glucose transport model, with a sampling frequency of 1 (starting from 1) time units.

Param./Factor	10^-2^	10^-1^	10^0^	10^1^	10^2^
*k*_1_	0.079/0.091	0.079/0.10	0.081/0.091	0.083/0.10	0.084/0.11
*k*_-1_	0.085/0.23	0.084/0.093	0.081/0.091	0.078/0.10	0.078/0.089
*k*_2_	0.16/0.93	0.080/0.55	0.081/0.091	0.063/0.083	0.054/0.062
*k*_-2_	0.054/0.10	0.062/0.074	0.081/0.091	0.097/0.10	0.10/0.10
*k*_3_	0.081/0.091	0.082/0.091	0.081/0.091	0.079/0.089	0.075/0.082
*k*_-3_	0.070/0.080	0.079/0.089	0.081/0.091	0.082/0.091	0.081/0.091
*k*_4_	0.23/0.32	0.21/0.30	0.081/0.091	6.36/6.93	310/336
*k*_-4_	310/336	6.36/6.93	0.081/0.091	0.21/0.30	0.23/0.32
*α*	0.10/0.10	0.095/0.17	0.081/0.091	0.080/0.089	0.083/0.088
*β*	0.076/0.088	0.091/0.096	0.081/0.091	0.075/0.15	0.074/0.19
All	0.16/0.97	0.081/0.69	0.081/0.091	0.060/0.091	0.054/0.081

The nominal parameter values *(k*_1 _= 1000, *k*_-1 _= 1100, *k*_2 _= 1000, *k*_-2 _= 1200, *k*_3 _= 1000, *k*_-3 _= 7000, *k*_4 _= 1000, *k*_-4 _= 1100, *α *= 4.2, and *β *= 1) are modified by a multiplicative factor, and the maximal (for any state variable) time average/infinity norm of the relative difference between the original and the reduced model is presented. Note that due to potential numerical inaccuracies only concentrations larger than 10^-6 ^are considered in the analysis.

## Discussion

In this paper we have presented a novel method for reduction of biochemical models that is compatible with the concept of zooming. Several methods for reduction of biochemical models already exist in the literature. However, few of these methods result in biochemically interpretable models, and to our knowledge there are no nonlinear lumping methods for which the state variables and parameters of the reduced model can be back-translated (mapped) to the original model.

The application of the QSS assumption has been a commonly used tool in the modeling of biochemical networks since the late 1960s, and in chemical kinetics for more than 80 years [38]. The validity of the QSS approximation is well studied both for specific biochemical mechanisms [38,39] and for more complex models [40,41]. The resulting equations, together with conservation relations, are typically used to eliminate some of the state variables in the model (e.g., see [40]). However, with the examples in this paper we have showed that such an approach is not always sufficient, and we propose to use proper lumping of state variables in combination with back-translation.

Our method has several important advantages when applied to biochemical models. The most important advantage is that we end up with reduced models with a clear biological interpretation, meaning that each state variable of the original model corresponds to a fraction of exactly one of the state variables in the reduced model. A consequence is that neighboring species in the original model remain neighbors in the reduced model. Hence we can consider the original and reduced models as two different degrees of zooming; a concept that we discussed in some detail in [27] for linear models.

The work in this paper can be seen as an extension of the theory introduced for linear models in [27] to nonlinear models. The method is based on assumptions regarding the dynamics that result in a sufficient number of equations that are linear w.r.t. the state variables to be back-translated. Such equations are typically a natural result of QSS assumptions and conservations relations in models based on mass action kinetics [42], and in particular in models that involve transporters and enzymes (e.g., the models in this paper). However, note that our method may also be applicable to models with other types of reaction kinetics. We also note that if too few linear relations are available for calculation of fraction parameters for a part of a model, this part can still be reduced and the reduced model can be simulated, although we cannot back-translate the corresponding modified lumped state variables since no fraction parameters are available. However, depending on the purpose of the model it may be enough to calculate fraction parameters for a subset of the state variables in the reduced model. Linearization of the model around a steady state operating point may also be a feasible approach to calculate fraction parameters with the method in [27].

The proposed method enables mapping of the state variables and parameters of the reduced model to those of the original model. In [27] we referred to this mapping as back-translation. Back-translation is of great importance, since we can directly observe how modifications to the reduced model impact the original model. It also gives the modeler an opportunity to check whether the assumptions underlying the reduction are acceptable. To illustrate the power of back-translation we provide plots for comparison of simulations of the original and reduced models, w.r.t. the original state variables, for the models to which the method is applied in this paper.

Back-translation of state variables typically requires the solution of a system of nonlinear equations, which often results from the assumption of state variables in QSS and conservation relations. Unfortunately, analytic solutions to systems of nonlinear equations do in general not exist. An advantage with the proposed method is that such solutions are not required, since they are replaced by computation of the inverse of a matrix for each cluster of fast state variables, which is in general a more feasible task.

Our method was applied to a small model with five state variables that commonly appears as part of larger biochemical models, and to a previously published model for the transport of glucose in baker's yeast (*S. cerevisiae*) [37]. The first model was reduced from five to one state variable, and from five to three parameters. However, note that our focus has been on the reduction of the number of state variables and not the number of parameters, which are reduced as a side-effect of the QSS assumptions.

The model for glucose transport was first reduced with an approach in which the QSS equations and conservation relations were directly substituted into the remaining ODEs. The results of this approach are not satisfactory since the reduced model gives predictions that are different from the original model for most state variables. Our method was then applied to the same model both together with the assumption of equal concentration of the transporter in the inner and outer regions of the cell membrane used in [37], and without any additional assumptions. The application of our method together with the assumptions used in [37] results in a model with three state variables. The state dynamics is significantly better preserved than with the first approach, although still not satisfactory. We then decided to reduce the original model without the assumption regarding the localization of the transporter, with two different definitions of modified lumped state variables. While one of these definitions results in a reduced model with four state variables and gave rather accurate predictions, the other choice reduces the number of state variables to five and gives an excellent description of the state dynamics. It is therefore apparent that there is a tradeoff between accuracy and the number of state variables in the reduction process. The glucose transport model corresponds to the first part of glycolysis, in which glucose is transported into the cell. We therefore propose that it might be rewarding to carefully re-investigate the assumptions underlying the reaction rate equations in complete models of glycolysis (see [43] for one example).

We have also observed a few issues regarding the implementation of the method. The symbolic inversion of the matrix that is necessary to compute the dynamics of the modified lumped state variables may be expensive. However, this is typically only a practical limitation for large matrices, which result from large clusters of fast state variables. In our experience large clusters of fast state variables are relatively rare also in large biochemical models. Another option, if it is not practically feasible to invert the symbolic matrix, is to solve the system of linear equations in Eq. (31) numerically. We also observed that the symbolic right-hand side of the resulting differential equations may be long. However, these are usually not practical limitations for the applicability of the method, e.g., the simulations of all examples in this paper are very fast on a modern computer. Available methods to reduce the analytic reaction rate expressions include sensitivity analysis w.r.t. state variables and parameters, and the method proposed in [17].

There is still no consensus method for automatic identification of state variables in QSS, although criteria for the detection of state variables in QSS have been proposed, for example in [30]. A simple approach is to simulate the original model and investigate for which state variables the corresponding in- and outflow reaction rates are approximately equal. State variables for which this condition holds are then considered to be in QSS. Note that for the models in this paper it was already clear from the biochemical understanding of the corresponding systems which of the state variables that could be considered fast (see [37] for the glucose transport example). However, an appropriate general criterion for automatic identification of state variables in QSS is still lacking.

Although the theory presented in this paper constitutes a great leap forward for construction of zoomable models, more research is required to make the method fully automatic. An important challenge is to define a meaningful measure for the similarity between the hierarchical model layers (degrees of zooming). Another interesting, although trivial, observation that deserves further attention is that QSS assumptions typically do not hold in the whole parameter space. Although the reduced models in this paper appear to be robust to varying parameter values it may not be the case in general. It may therefore be revealing to compare the original model and the reduced model to characterize the parameter space regions in which the QSS-assumptions are valid.

## Conclusions

We have presented a novel method for reduction of biochemical models that is compatible with the concept of zooming. Zooming allows the modeler to operate on different levels of model granularity, and enables a direct interpretation of how modifications to the model on one level affect the same model on other levels in the hierarchy. The proposed method is based on the application of proper lumping in combination with the identification of linear relations in nonlinear equations.

The method was applied to two example models. The first model is small and commonly occurring as a part of larger biochemical models. The second example is a model for glucose transport in baker's yeast, which constitutes the starting point for glycolysis. Both models could be significantly reduced with the proposed method, and the resulting state variables could be back-translated to the original state variables. The method that is presented in this paper constitutes an extension of the method that was previously developed for linear biochemical models to its nonlinear counterpart. Since most models in the systems biology community are in fact nonlinear, our method constitutes an important step towards zoomable biochemical models.

## Authors' contributions

MS developed the theory, most of the aspects of the method, and did the calculations within this project, which was managed by MJ. MS wrote the major part of the paper, with contributions from GC and MJ. All authors read and approved the final manuscript.

## A Appendix

### A.1 Appendix 1

The ordinary differential equations for the enzyme kinetics model take the form

$$\begin{array}{llll} ẋ & =\left(\begin{array}{c} Ṡ\\ Ė\\ Ṗ\\ \overset{{}^{\circ}}{C_{S}}\\ \overset{{}^{\circ}}{C_{P}} \end{array}\right)=\left(\begin{array}{c} -k_{1}SE+k_{-1}C_{S}\\ -k_{1}SE+k_{-1}C_{S}+k_{3}C_{P}-k_{-3}PE\\ k_{3}C_{P}-k_{-3}PE\\ k_{1}SE-k_{-1}C_{S}-k_{2}C_{S}\\ k_{2}C_{S}-k_{3}C_{P}+k_{-3}PE \end{array}\right)=\; & & \;\\ & =\left(\begin{array}{ccc} -1 & 0 & 0\\ -1 & 0 & 1\\ 0 & 0 & 1\\ 1 & -1 & 0\\ 0 & 1 & -1 \end{array}\right)\left(\begin{array}{c} r_{1}\\ r_{2}\\ r_{3} \end{array}\right)=Sr\left(x,p\right),\; & & \; \end{array}$$

where *r*_1 _= *k*_1_*SE *- *k*_-1_*C_S_*, *r*_2 _= *k*_2_*C_S_*, and *r*_3 _= *k*_3_*C_P _*- *k*_-3_*PE*.

The parameters are set to values that satisfy the assumptions of dominating and insignificant reaction terms, with *k*_1 _= 1000, *k*_-1 _= 2000, *k*_2 _= 1, *k*_3 _= 3000, and *k*_-3 _= 1000, together with the initial conditions *S*(0) = *E*(0) = 1 and *P*(0) = *C_S_*(0) = *C_P_*(0) = 0. This gives the parameter values *M*_1 _= 0.5, *M*_3 _= 3 and *L_E _*= 1 in the reduced model. Initial conditions can in general be obtained from a short simulation of the original model, until the fast state variables reach QSS, but in this case Eq. (19) gives an analytic expression of *S*(0) (*P*(0) = 0)

$$\begin{array}{llll} S\left(0\right) & =\frac{1}{2}\left(L_{S}-L_{E}-M_{1}^{-1}+\sqrt{{\left(L_{S}+L_{E}+M_{1}^{-1}\right)}^{2}-4L_{S}L_{E}}\right)=\; & & \;\\ & =\sqrt{3}-1,\; & & \; \end{array}$$

where $M_{1}^{-1}=K_{1}$, *S *= *A*, *L_S _*= *L*_1_, and *L_E _*= *L*_2_.

### A.2 Appendix 2

The ordinary differential equations for the glucose transport model take the form

$$\begin{array}{llll} \frac{dx_{Glc}^{e}}{dt} & =-k_{1}x_{E}^{e}x_{Glc}^{e}+k_{-1}x_{E-Glc}^{e},\; & & \;\\ \frac{dx_{Glc}^{i}}{dt} & =-k_{2}x_{E}^{i}x_{Glc}^{i}+k_{-2}x_{E-Glc}^{i},\; & & \;\\ \frac{dx_{E-G6P}^{i}}{dt} & =k_{4}x_{E}^{i}x_{G6P}^{i}-k_{-4}x_{E-G6P}^{i},\; & & \;\\ \frac{dx_{E-Glc-G6P}^{i}}{dt} & =k_{3}x_{E-Glc}^{i}x_{G6P}^{i}-k_{-3}x_{E-Glc-G6P}^{i},\; & & \;\\ \frac{dx_{G6P}^{i}}{dt} & =-k_{3}x_{E-Glc}^{i}x_{G6P}^{i}+k_{-3}x_{E-Glc-G6P}^{i}-\; & & \;\\ & k_{4}x_{E}^{i}x_{G6P}^{i}+k_{-4}x_{E-G6P}^{i},\; & & \;\\ \frac{dx_{E-Glc}^{e}}{dt} & =\alpha \left(x_{E-Glc}^{i}-x_{E-Glc}^{e}\right)+k_{1}x_{E}^{e}x_{Glc}^{e}-k_{-1}x_{E-Glc}^{e},\; & & \;\\ \frac{dx_{E-Glc}^{i}}{dt} & =\alpha \left(x_{E-Glc}^{e}-x_{E-Glc}^{i}\right)-k_{3}x_{E-Glc}^{i}x_{G6P}^{i}+\; & & \;\\ & k_{-3}x_{E-Glc-G6P}^{i}+k_{2}x_{E}^{i}x_{Glc}^{i}-k_{-2}x_{E-Glc}^{i},\; & & \;\\ \frac{dx_{E}^{e}}{dt} & =\beta \left(x_{E}^{i}-x_{E}^{e}\right)-k_{1}x_{E}^{e}x_{Glc}^{e}+k_{-1}x_{E-Glc}^{e},\; & & \;\\ \frac{dx_{E}^{i}}{dt} & =\beta \left(x_{E}^{e}-x_{E}^{i}\right)-k_{4}x_{E}^{i}x_{G6P}^{i}+k_{-4}x_{E-G6P}^{i}-\; & & \;\\ & k_{2}x_{E}^{i}x_{Glc}^{i}+k_{-2}x_{E-Glc}^{i},\; & & \; \end{array}$$

which was introduced in [27].

### A.3 Appendix 3

In this section we investigate an alternative (naive) approach to reduce the glucose transport model. The first step is to identify state variables for which the QSS assumption holds, and the mass conservation relations in the model. In the second step of this approach we then substitute the corresponding system of equations into the ODEs corresponding to slow state variables.

Now consider the model for glucose transport in yeast. We assume that the state variables $x_{Glc}^{e}$, $x_{Glc}^{i}$, $x_{E-G6P}^{i}$ and $x_{E-Glc-G6P}^{i}$ are in QSS, which gives Eqs. (43)-(46). Note that Eqs. (45)-(46) indirectly imply that $x_{G6P}^{i}$ is in steady state. The substitution of Eqs. (43) - (46) into the ODEs of the original model gives

(61) $$\frac{dx_{E-Glc}^{e}}{dt}\approx \alpha \left(x_{E-Glc}^{i}-x_{E-Glc}^{e}\right),$$

(62) $$\frac{dx_{E-Glc}^{i}}{dt}\approx \alpha \left(x_{E-Glc}^{e}-x_{E-Glc}^{i}\right),$$

(63) $$\frac{dx_{E}^{e}}{dt}\approx \beta \left(x_{E}^{i}-x_{E}^{e}\right),$$

(64) $$\frac{dx_{E}^{i}}{dt}\approx \beta \left(x_{E}^{e}-x_{E}^{i}\right).$$

Note that the state variables $x_{E-Glc}^{e}$ and $x_{E-Glc}^{i}$ are decoupled from the state variables $x_{E}^{e}$ and $x_{E}^{i}$ in Eqs. (61)-(64).

There are three molecules (moieties) whose mass is conserved in the model as a whole, i.e., Glc, G6P, and E. However, we can not substitute any of the conservation relations into the remaining ODEs without re-introducing state variables that were already eliminated. So the final reduced model takes the form of Eqs. (61) - (64). However, the sum of the state variables $x_{E-Glc}^{e}$ and $x_{E-Glc}^{i}$, and $x_{E}^{e}$ and $x_{E}^{i}$ is conserved in the reduced model, which makes it possible to reduce the model to two state variables.

Unfortunately, due to the form of the ODEs and the initial conditions of the state variables in the reduced model, the state variables $x_{E-Glc}^{e}$ and $x_{E-Glc}^{i}$ remain equal to zero at all times, and only the state variables $x_{E}^{e}$ and $x_{E}^{i}$ take non-zero values. We therefore decided to simulate the original model for a short time until the fast state variables reach QSS, and to use the final state variable values as initial conditions in the reduced model.

The solution to the equation system defined by Eqs. (43)-(46) and Eq. (49) is

$$\begin{array}{c} x_{Glc}^{e}\approx K_{1}\frac{x_{E-Glc}^{e}}{x_{E}^{e}},\\ x_{Glc}^{i}\approx K_{2}\frac{x_{E-Glc}^{i}}{x_{E}^{i}},\\ x_{G6P}^{i}\approx \frac{K_{3}K_{4}}{K_{4}x_{E-Glc}^{i}+K_{3}x_{E}^{i}+K_{3}K_{4}}L_{G6P},\\ x_{E-G6P}^{i}\approx \frac{K_{3}x_{E}^{i}}{K_{4}x_{E-Glc}^{i}+K_{3}x_{E}^{i}+K_{3}K_{4}}L_{G6P},\\ x_{E-Glc-G6P}^{i}\approx \frac{K_{4}x_{E-Glc}^{i}}{K_{4}x_{E-Glc}^{i}+K_{3}x_{E}^{i}+K_{3}K_{4}}L_{G6P}, \end{array}$$

which can be used for back-translation of the state variables of the reduced model to those of the original model.

The predictions of the state variables of the original model, resulting from simulations of the original model and the reduced model with the parameter values set as in [37], is not satisfactory for any other state variable than *G*6*P*, which remains approximately constant over time. Implementations of the original model and the reduced model in SBtoolbox2 for MATLAB [36] are included in *Additional files*.

### A.4 Appendix 4

In this section we apply our method to the glucose transport model, and following [37] we will assume that the concentrations of the transporter are constant in the inner and outer regions of the cellular membrane. With this assumption the distribution among the transporter state variables of the original model, which constitute the lumped state variables *L_E_*, is uniquely defined.

The first step of the method is to identify the apparent conservation relations in the model. We note that G6P and the transporter E are conserved, and apparent conserved glucose (see Definition 1) in the inner and outer regions of the membrane, respectively. The four apparent conservation relations take the form

$$\begin{array}{llll} l & =\left(\begin{array}{c} L_{Glc_{1}}\\ L_{Glc_{2}}\\ L_{G6P}\\ L_{E} \end{array}\right)=Mx=\; & & \;\\ & =\left(\begin{array}{ccccccccc} 1 & 0 & 0 & 0 & 0 & 1 & 0 & 0 & 0\\ 0 & 1 & 0 & 1 & 0 & 0 & 1 & 0 & 0\\ 0 & 0 & 1 & 1 & 1 & 0 & 0 & 0 & 0\\ 0 & 0 & 1 & 1 & 0 & 1 & 1 & 1 & 1 \end{array}\right)x,\; & & \; \end{array}$$

where $x={\left(x_{Glc}^{e}\;x_{Glc}^{i}\;x_{E-G6P}^{i}\;x_{E-Glc-G6P}^{i}\;x_{G6P}^{i}\;x_{E-Glc}^{e}\;x_{E-Glc}^{i}\;x_{E}^{e}\;x_{E}^{i}\right)}^{T}$.

In the second step of the method we define the modified lumped state variables. We decide to keep *L_E _*in the reduced model since it corresponds to an exact conservation, and therefore results in the largest reduction possible (note that the exact conservation relations, *L_E _*and *L*_*G*6*P*_, can not simultaneously be used since the lumping would then not be proper). The modified lumped state variables take the form

$$\begin{array}{llll} l_{m} & =\left(\begin{array}{c} x_{Glc}^{e}\\ x_{Glc}^{i}\\ x_{G6P}^{i}\\ L_{E} \end{array}\right)=M_{m}x=\; & & \;\\ & =\left(\begin{array}{ccccccccc} 1 & 0 & 0 & 0 & 0 & 0 & 0 & 0 & 0\\ 0 & 1 & 0 & 0 & 0 & 0 & 0 & 0 & 0\\ 0 & 0 & 0 & 0 & 1 & 0 & 0 & 0 & 0\\ 0 & 0 & 1 & 1 & 0 & 1 & 1 & 1 & 1 \end{array}\right)x.\; & & \; \end{array}$$

We note that Eqs. (43)-(47), and (50) are all linear w.r.t. the state variables that constitute state *L_E_*, so the requirement for the existence of at least 6 (*n_m_*) linear relations is satisfied, which enables back-translation in step three of the method.

In the third step of the method we derive the fraction parameters for the lumped state variable *L_E_*. Eqs. (43)-(47), and (50) form an equation system, corresponding to Eq. (28)

(65) $$\begin{array}{lll} A\left(l_{m},p\right)x_{m_{k}} & =\left(\begin{array}{cccccc} 0 & 0 & 1 & 0 & -x_{Glc}^{e^{\prime }} & 0\\ 0 & 0 & 0 & 1 & 0 & -x_{Glc}^{i^{\prime }}\\ 1 & 0 & 0 & 0 & 0 & -x_{G6P}^{i^{\prime }}\\ 0 & 1 & 0 & -x_{G6P}^{i”} & 0 & 0\\ 0 & 0 & \alpha & -\alpha & \beta & -\beta \\ 1 & 1 & 1 & 1 & 1 & 1 \end{array}\right)\cdot \; & \text{(1)}\\ & \cdot \left(\begin{array}{c} x_{E-G6P}^{i}\\ x_{E-Glc-G6P}^{i}\\ x_{E-Glc}^{e}\\ x_{E-Glc}^{i}\\ x_{E}^{e}\\ x_{E}^{i} \end{array}\right)\approx \left(\begin{array}{c} 0\\ 0\\ 0\\ 0\\ 0\\ x_{L}^{\prime } \end{array}\right)=b_{k}\left(l_{m},p\right),\; & \text{(2)} \end{array}$$

where $x_{Glc}^{e^{\prime }}=\frac{k_{1}}{k_{-1}}x_{Glc}^{e}$, $x_{Glc}^{i^{\prime }}=\frac{k_{2}}{k_{-2}}x_{Glc}^{i}$, $x_{G6P}^{i^{\prime }}=\frac{k_{2}}{k_{-2}}x_{G6P}^{i}$ and $x_{G6P}^{i}=\frac{k_{4}}{k-4}x_{G6P}^{i}$. The solution to Eq. (65) is given by Eq. (29)

(66) $$\begin{array}{lll} \left(\begin{array}{c} x_{E-G6P}^{i}\\ x_{E-Glc-G6P}^{i}\\ x_{E-Glc}^{e}\\ x_{E-Glc}^{i}\\ x_{E}^{e}\\ x_{E}^{i} \end{array}\right) & =\left(\begin{array}{c} \eta_{E-G6P}^{i}\\ \eta_{E-Glc-G6P}^{i}\\ \eta_{E-Glc}^{e}\\ \eta_{E-Glc}^{i}\\ \eta_{E}^{e}\\ \eta_{E}^{i} \end{array}\right)L_{E}\approx \; & \text{(1)}\\ \approx & \left(\begin{array}{c} \frac{x_{G6P}^{i^{\prime }}\left(\beta +\alpha x_{Glc}^{e^{\prime }}\right)}{\zeta }\\ \frac{x_{G6P}^{i”}x_{Glc}^{ip}\left(\beta +\alpha x_{Glc}^{e^{\prime }}\right)}{\zeta }\\ \frac{x_{Glc}^{e^{\prime }}\left(\beta +\alpha x_{Glc}^{i^{\prime }}\right)\left(\beta +\alpha x_{Glc}^{e^{\prime }}\right)}{\zeta }\\ \frac{x_{Glc}^{i^{\prime }}\left(\beta +\alpha x_{Glc}^{e^{\prime }}\right)}{\zeta }\\ \frac{\left(\beta +\alpha x_{Glc}^{i^{\prime }}\right)\left(\beta +\alpha x_{Glc}^{e^{\prime }}\right)}{\zeta }\\ \frac{\left(\beta +\alpha x_{Glc}^{e^{\prime }}\right)}{\zeta } \end{array}\right)L_{E}\; & \text{(2)} \end{array}$$

where

(67) $$\begin{array}{l} \zeta =(\beta x_{Glc}^{e^{\prime }}+2x_{Glc}^{e^{\prime }}\alpha x_{Glc}^{i^{\prime }}+2\beta +\alpha x_{Glc}^{i^{\prime }}+\alpha x_{Glc}^{e^{\prime }}+\beta x_{Glc}^{i^{\prime }}x_{G6P}^{i}+\cdots \\ +\alpha x_{Glc}^{i^{\prime }}x_{G6P}^{i}x_{Glc}^{e^{\prime }}+\beta x_{Glc}^{i^{\prime }}+\beta x_{G6P}^{i^{\prime }}+\alpha x_{Glc}^{e^{\prime }}x_{G6P}^{i^{\prime }}) \end{array}$$

and where the fraction parameters were calculated with Eq. (30). We note that the fraction parameters are functions of $x_{Glc}^{e}$, $x_{Glc}^{i}$, and $x_{G6P}^{i}$, which are state variables both in the original- and in the reduced model. In the fourth step of the method we derive differential equations for the modified lumped state variables. The ODE for the fourth state is ${\overset{{}^{\circ}}{l}}_{m_{4}}={\overset{{}^{\circ}}{L}}_{E}=0$, which is replace by a constant. The ODEs for the other states are

$${\overset{{}^{\circ}}{l}}_{m_{1:3}}=\left(\begin{array}{c} {ẋ}_{Glc}^{e}\\ {ẋ}_{Glc}^{i}\\ {ẋ}_{G6P}^{i} \end{array}\right)={\left(\begin{array}{ccc} 1+J_{11} & J_{12} & J_{13}\\ J_{21} & 1+J_{22} & J_{23}\\ J_{31} & J_{32} & 1+J_{33} \end{array}\right)}^{-1}{\overset{{}^{\circ}}{l}}_{1:3}$$

where $l_{m_{1:3}}$ denotes the first three states variables in *l_m_*. Note that there are three state variables in the reduced model, which is the same number as for the reduced model in [37].

In the fifth step of our method we compare predictions of the original state variables between the original and reduced models, where *L_E _*is back-translated with the fraction parameters defined in Eqs. (66)-(67).

The simulation results are clearly more accurate than with the approach in Appendix A.3, although still not satifying. We refer to *Additional files *for implementations of the original and reduced models in SBtoolbox2 for MATLAB [36].

### A.5 Appendix 5

In this section we apply our method to the glucose transport model, but with an alternative definition of the modified lumped state variables. We do not use the assumption of constant regional concentrations of the transporter (Eq. (50)).

In the first step of the method we note that the apparent conservations are given by Eq. (51).

In the second step of our method we decide to keep state variable $L_{E_{2}}$, instead of *L*_*G*6*P*_, in the reduced model. This leads to the following definition of the modified lumped state variables

(68) $$\begin{array}{llll} l_{m} & =\left(\begin{array}{c} x_{Glc}^{e}\\ L_{E_{1}}\\ x_{Glc}^{i}\\ x_{G6P}^{i}\\ L_{E_{2}} \end{array}\right)=M_{m}x=\; & \text{(1)}\\ & =\left(\begin{array}{ccccccccc} 1 & 0 & 0 & 0 & 0 & 0 & 0 & 0 & 0\\ 0 & 0 & 0 & 0 & 0 & 1 & 0 & 1 & 0\\ 0 & 1 & 0 & 0 & 0 & 0 & 0 & 0 & 0\\ 0 & 0 & 0 & 0 & 1 & 0 & 0 & 0 & 0\\ 0 & 0 & 1 & 1 & 0 & 0 & 1 & 0 & 1 \end{array}\right)x,\; & & \; \end{array}$$

Where $x={\left(x_{Glc}^{e}\;x_{Glc}^{i}\;x_{E-G6P}^{i}\;x_{E-Glc-G6P}^{i}\;x_{G6P}^{i}\;x_{E-Glc}^{e}\;x_{E-Glc}^{i}\;x_{E}^{e}\;x_{E}^{i}\right)}^{T}$, and we note that $x_{Glc}^{e}$, $x_{Glc}^{i}$, and $x_{G6P}^{i}$ are state variables both in the original and reduced models. Also note that the requirement of at least *n_m _*equations, that are linear w.r.t. the original state variables and linearly independent, is satified for each of the modified lumped state variables by Eqs. (43)-(49).

In the third step of the method we calculate fraction parameters for the modified lumped state variables $L_{E_{1}}$ and $L_{E_{2}}$, which correspond to more than one of the original state variables. The fraction parameters for state variable $L_{E_{1}}$ are given by Eq. (53). We can now use Eqs. (44)-(46) and Eq. (68) to form an equation system corresponding to Eq. (28)

$$\begin{array}{llll} A\left(l_{m},p\right)x_{m_{k}} & =\left(\begin{array}{cccc} -x_{Glc}^{i} & K_{2} & 0 & 0\\ -x_{G6P}^{i} & 0 & K_{4} & 0\\ 0 & -x_{G6P}^{i} & 0 & K_{3}\\ 1 & 1 & 1 & 1 \end{array}\right)\left(\begin{array}{c} x_{E}^{i}\\ x_{E-Glc}^{i}\\ x_{E-G6P}^{i}\\ x_{E-Glc-G6P}^{i} \end{array}\right)\approx \; & & \;\\ \approx & \left(\begin{array}{c} 0\\ 0\\ 0\\ L_{E_{2}} \end{array}\right)=b_{k}\left(l_{m},p\right),\; & & \; \end{array}$$

with the solution given by Eq. (29)

(69) $$\begin{array}{llll} \left(\begin{array}{c} x_{E}^{i}\\ x_{E-Glc}^{i}\\ x_{E-G6P}^{i}\\ x_{E-Glc-G6P}^{i} \end{array}\right) & =\left(\begin{array}{c} \eta_{E}^{i}\\ \eta_{E-Glc}^{i}\\ \eta_{E-G6P}^{i}\\ \eta_{E-Glc-G6P}^{i} \end{array}\right)L_{E_{2}}\approx \; & \text{(1)}\\ \approx & \left(\begin{array}{c} \frac{K_{2}K_{3}K_{4}}{\xi }\\ \frac{K_{3}K_{4}x_{Glc}^{i}}{\xi }\\ \frac{K_{2}K_{3}x_{G6P}^{i}}{\xi }\\ \frac{K_{4}x_{G6P}^{i}x_{Glc}^{i}}{\xi } \end{array}\right)L_{E_{2}},\; & & \; \end{array}$$

where

$$\xi =x_{G6P}^{i}\left(K_{4}x_{Glc}^{i}+K_{2}K_{3}\right)+K_{3}K_{4}x_{Glc}^{i}+K_{2}K_{3}K_{4}$$

and the fraction parameters were calculated with Eq. (30).

The fourth step of the method is to derive ODEs for the modified lumped state variables. Since the apparent conservations are separated into two disjoint clusters of fast state variables, we can treat the model for the inner and outer regions of the membrane separately. The rate equations for the outer region are given by Eqs. (56)-(57). Eq. (31) gives us the ODEs of the modified lumped state variables in the inner region

(70) $$\begin{array}{llll} {\overset{{}^{\circ}}{l}}_{m_{3:5}} & =\left(\begin{array}{c} {ẋ}_{Glc}^{i}\\ {ẋ}_{G6P}^{i}\\ {\overset{{}^{\circ}}{L}}_{E_{2}} \end{array}\right)={\left(I+J\right)}^{-1}\overset{{}^{\circ}}{l}=\; & \text{(1)}\\ & ={\left(\begin{array}{ccc} 1+J_{33} & J_{34} & J_{35}\\ J_{43} & 1+J_{44} & J_{45}\\ 0 & 0 & 1 \end{array}\right)}^{-1}{\overset{{}^{\circ}}{l}}_{3:5},\; & & \; \end{array}$$

where *l*_3:5 _and $l_{m_{3:5}}$ are the last three state variables of *l *and *l_m_*, respectively, and

(71) $$\begin{array}{llll} {\overset{{}^{\circ}}{l}}_{3:5} & =\left(\begin{array}{c} {\overset{{}^{\circ}}{L}}_{Glc_{2}}\\ {\overset{{}^{\circ}}{L}}_{G6P}\\ {\overset{{}^{\circ}}{L}}_{E_{2}} \end{array}\right)\approx \; & \text{(1)}\\ & \approx \left(\begin{array}{c} -\alpha \left(x_{E-Glc}^{i}-x_{E-Glc}^{e}\right)\\ 0\\ -\alpha \left(x_{E-Glc}^{i}-x_{E-Glc}^{e}\right)-\beta \left(x_{E}^{i}-x_{E}^{e}\right) \end{array}\right),\; & & \; \end{array}$$

In the fifth step of the method we simulate the reduced model with Eqs. (56)-(57) and (70)-(71) and we then use Eqs. (53) and (69) for back-translation of the state variables. A comparison between the original model and the reduced model, w.r.t. the state variables of the original model, is presented in Figure 5 and Figure 6. The agreement between the models is very good. We refer to *Additional files *for implementations of the original and reduced models in SBtoolbox2 for MATLAB [36].

## Additional files

The original and reduced versions of the models presented in this paper, and scripts for simulation and comparison between the original and reduced versions of the models. Note that the systems biology toolbox for MATLAB [36] and the symbolic math toolbox for MATLAB must be installed on the system for simulation of the attached models.

## Supplementary Material

Additional file 1 **Model 1**. The original enzyme kinetics model. Click here for file

Additional file 2 **Model 2**. The reduced enzyme kinetics model. Click here for file

Additional file 3 **Script 1**. Script for comparison between the original enzyme kinetics model and the reduced model. Click here for file

Additional file 4 **Model 3**. The original glucose transport model. Click here for file

Additional file 5 **Model 4**. The reduced glucose transport model with the alternative (naive) approach. Click here for file

Additional file 6 **Script 2**. Script for comparison between the original glucose transport model and the reduced model with the alternative (naive) approach. Click here for file

Additional file 7 **Model 5**. The reduced glucose transport model with our method and the assumption of constant concentrations of the transporter in the inner and outer regions of the cellular membrane. Click here for file

Additional file 8 **Script 3**. Script for comparison between the original glucose transport model and the reduced model with our method and the assumption of constant concentrations of the transporter in the inner and outer regions of the cellular membrane. Click here for file

Additional file 9 **Model 6**. The reduced glucose transport model with four state variables with our method. Click here for file

Additional file 10 **Script 4**. Script for comparison between the original glucose transport model and the reduced model with four state variables with our method. Click here for file

Additional file 11 **Model 7**. The reduced glucose transport model with five state variables with our method. Click here for file

Additional file 12 **Script 5**. Script for comparison between the original glucose transport model and the reduced model with five state variables with our method. Click here for file
